# The biology of bone lengthening

**DOI:** 10.1007/s11832-016-0780-2

**Published:** 2016-11-12

**Authors:** Ivan Hvid, Joachim Horn, Stefan Huhnstock, Harald Steen

**Affiliations:** Section of Pediatric and Reconstructive Orthopaedic Surgery, Department of Orthopaedic Surgery, Oslo University Hospital, Rikshospitalet, Sognsvannsveien 20, 0372 Oslo, Norway

**Keywords:** Bone, Lengthening, Biology, Stem cells, Platelets, Growth factors

## Abstract

Distraction osteogenesis biologically resembles fracture healing with distinctive characteristics notably in the distraction phase of osteogenesis. In the latency phase of bone lengthening, like in the inflammatory phase of fracture repair, interleukines are released and act with growth factors released from platelets in the local haematoma, leading to attraction, proliferation and differentiation of mesenchymal stem cells into osteoblasts and other differentiated mesenchymal cells. These in turn produce matrix, collagen fibers and growth factors. A callus containing cells, collagen fibers, osteoid and cartilage matrix is formed. Provided stable fixation, distraction will trigger intramembranous bone formation. As distraction proceeds, the distraction gap develops five distinctive zones with unmineralized bone in the middle, remodelling bone peripherally, and mineralizing bone in between. During consolidation, the high concentration of anabolic growth factors in the regenerate diminishes with time as remodelling takes over to form mature cortical and cancellous bone. Systemic disease, congenital bone deficiencies, medications and substance abuse can influence the quality and quantity of regenerate bone, usually in a negative way. The regenerate bone can be manipulated when needed by using injection of mesenchymal stem cells and platelets, growth factors (BMP-2 and -7), and systemic medications (bisphosphonates and parathyroid hormone). Growth factors and systemic anabolic and antiresorptive drugs are prescribed on special indications, while distraction osteogenesis is not an authorized indication. To some extent, however, these compounds can be used off-label. Use in children presents special problems since growth factors and specific anabolic medications may involve a risk of inducing cancer.

## Introduction

Optimal conditions for the lengthening of long bones are well established. The basic requirements for an optimal result of bone lengthening are minimally traumatic osteotomies preferably located in a metaphysis, solid mechanical fixation across osteotomies, an adequate latency period before lengthening to establish repair processes, suitable rhythm and amplitude of lengthening, a realistic goal regarding the extent of lengthening, and sufficient time for callus to mature before frame or nail removal [1]. Despite observation of all these requirements, in some instances bone formation can be insufficient, and additional procedures may be required to obtain a stable regenerate. Knowledge of the biological processes involved in distraction osteogenesis, and of diseases influencing these processes (e.g. diabetes mellitus), provides the clinician with tools to deal with the relatively infrequent problems related to underlying disease, and to problems related to the quantity and quality of bone formed during and after distraction.

## Distraction osteogenesis

The clinical method of bone lengthening is often referred to as distraction osteogenesis or callus distraction (callotasis), a term suggesting that fracture repair mechanisms are fundamental to initiate bone formation, and that distraction of the osteotomies will maintain these mechanisms for some time [2]. The initial phase of fracture healing, the inflammatory phase, is biologically identical to the initial phase of distraction osteogenesis, the latency phase [3]. The distraction phase of bone lengthening has distinguishing features from fracture healing, notably in the mode of ossification, intramembranous bone formation being predominant [3]. The final stage of fracture healing involving consolidation of the fracture and remodelling of the callus again is similar to the consolidation phase in distraction osteogenesis.

### The latency phase

The minimally invasive, low energy osteotomy can be compared to a closed low energy fracture. Secure fixation using an external fixator system or, in the older child or adult, an intramedullary lengthening device, should ensure an optimal local environment for the initial healing response. The length of the latency chosen is usually from 3 to 10 days, depending on the age of the patient, the site of lengthening (e.g. proximal tibial lengthening will benefit from a longer latency period compared to a distal femur), underlying disease and diagnosis [e.g. lengthening in congenital limb length discrepancy (LLD) may benefit from a longer latency period compared to acquired LLD], local factors such as previous infection, irradiation or a poor soft tissue envelope, ongoing pharmacotherapy such as NSAID’s or steroids, and environmental factors such as smoking (parental smoking could be an issue).

**Fig. 1:**
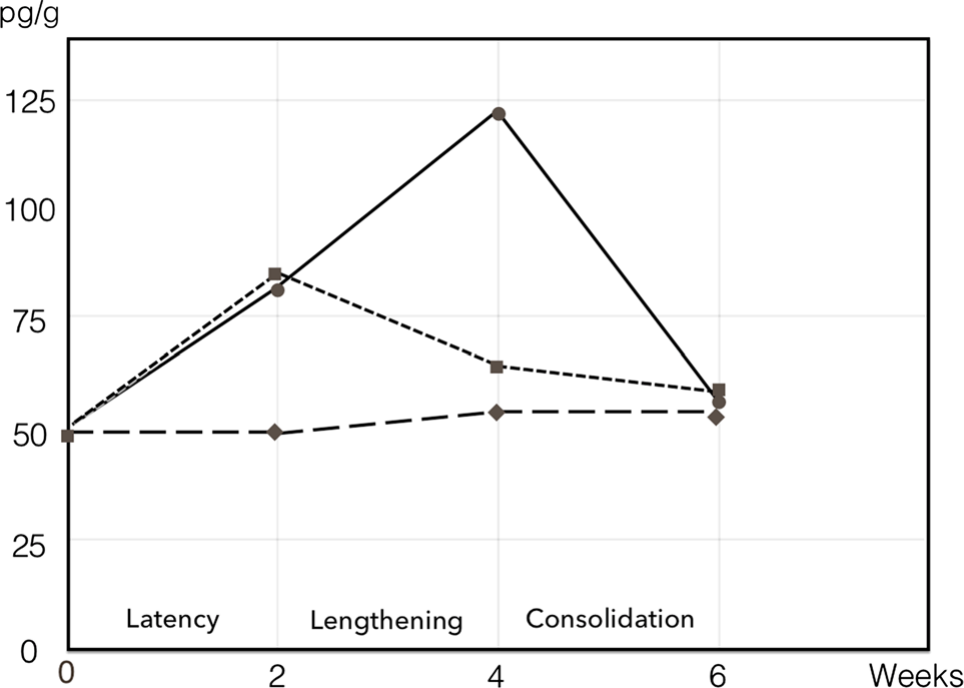
IGF-1 in periosteum as a function of time after osteotomy of the tibial shaft in a rabbit model. Mean data for non-osteotomized control tibiae (*filled diamond*), osteotomized control tibiae (*filled square*), and lengthened tibiae (*filled circle*) are shown. Phases of lengthening are indicated. Osteotomy and lengthening values different from control values at 2 weeks, lengthening values different from osteotomy and control values at 4 weeks (*P* < 0.05) Data obtained from [2], tables 1–3

The local trauma of osteotomy induces an inflammatory response causing release of cytokines, notably interleukines (IL-1, IL-6), leading to the recruitment, proliferation and differentiation of mesenchymal stem cells (MSCs or osteoprogenitor cells differentiate into osteoblasts) from bone marrow, periosteum and endosteum. These cells produce a variety of growth factors (GF) including bone morphogenetic proteins (BMPs, notably BMP-2, BMP-4 and BMP-6), transforming growth factor β (TGF-b), platelet derived growth factor (PDGF), and insulin-like growth factor (IGF-1) (Fig. 1) [2–5]. The haematoma formed in the osteotomy gap and its immediate surroundings will be inhabited by fibroblasts, chondroblasts and osteoblasts. In fracture repair, bone formation proceeds through callus formation, bone being formed mostly through endochondral bone formation when there is some instability across the fracture site, and mostly through intramembranous bone formation when there is a more stable fixation across a minimal fracture gap.

### The distraction phase

After latency, the osteotomy gap is distracted using a set rhythm and amplitude. The amount of lengthening per day is usually set at 1 mm at increments of 0.25 mm 4 times per day. This has proved a practical compromise. Increasing the rhythm (decreasing increments) leads to better bone formation [6].

Distraction modifies the fracture healing process influencing mainly the level and timing of expression of growth factors and other mediators [3]. This mechanotransduction creates an ordered network of bone collagen. Initially, this collagen tends to be a mixture of collagen type I (bone collagen) and type II (cartilage collagen), but soon after distraction is commenced, collagen type I predominates entirely. Bone matrix proteins are secreted, and mineral is incorporated to produce bone [7]. Mineralisation can usually be seen on ordinary radiographs after 1–2 weeks of distraction. Collagen fibers and cells align themselves along the direction of tensile strain. Typically, five zones can be distinguished between the native bone ends: a central fibrous zone, two peripheral mineralized zones, and two intermediate zones containing mineralizing microcolumns [3].

The growth factors appearing during the latency phase are still in play, their amounts peaking during the distraction phase. Other growth factors appear. Vascular endothelial growth factor (VEGF) and angiopoitin 1 and 2 promote the establishment of a capillary network. Remodelling is initiated quite early on, reflected by an increasing ratio of RANK/OPG [RANK is receptor activator of nuclear factor-kappaβ that will recruit osteoclasts, OPG is osteoprotegerin which protects bone from excessive resorption by binding to RANKL (RANK ligand)]. An increased ratio means that more osteoclasts are formed and activated thus setting the stage for the well known cyclus of bone remodelling [8].

Parathyroid hormone (PTH) plays a central role in the process of mineralisation which is also dependent on availability of vitamin D and calcium. It also influences the RANKL/RANK/OPG signalling system as a systemic regulator of bone remodelling [9]. It interplays with the Wnt signalling pathway (read “wint”, the name refers to its primary discovery in fruit flies—wingless) [10, 11] which profoundly influences bone formation and remodelling throughout embryonic and postnatal life. IGF-1 is essential to the actions of PTH on the skeleton, including the effects it might have in bone regenerative interventions [2, 12, 13]. IGF-1 regulates osteoblast function in endocrine (produced in the liver through growth hormone stimulation, acting on many tissues including bone), paracrine (influencing cells in the local environment) and autocrine (modifying the metabolism of the cell in which it is produced) fashions. It is part of a complicated system including 6 IGF binding proteins (BPs), some of which stimulate IGF-1 actions (IGFBP 3 and 5), and some of which inhibits its actions.

The upper limit of length gain in distraction osteogenesis of long bones is related to complications including soft tissue problems. There is no absolute consensus on this issue, but most authors would agree on 7–8 cm in the femur and 6–7 cm in the lower leg. We are unaware of specific biological restraints to indicate an upper limit to the gain in length that might be achieved.

### The consolidation phase

By the end of the distraction phase, the regenerate bone features a central unmineralized zone, neighbouring zones of mineralizing tissue, and peripheral zones consisting of wowen bone already influenced by the remodelling processes. As the central parts of the regenerate evolves into bone, the growth factors peaking during the distraction phase are downregulated, and remodelling takes over. PTH and the Wnt signalling pathways are important in this phase. The RANK/OPG ratio decreases, and TNF-α (tumor necrosis factor, induces apoptosis) is upregulated. Intermittent mechanical stimulation is likely to play a role in maintaining bone mass of the regenerate bone during this phase of active remodelling, underscoring the importance of keeping patients mobile and weightbearing. Active weightbearing also has a positive effect during the distraction phase, and it serves to minimize loss of bone mineral in the native bone being lengthened [14].

## Influence of disease, medication and substance abuse

Diabetes mellitus is reported to have a negative effect on bone regeneration, more so in type I than in type II diabetes. Insulin has a direct effect on osteogenesis through the stimulation of osteoblast differentiation, an effect not to be confused with that of IGF-1, although the two are phylogenetically related and may have some interactions [15]. Diabetes significantly affects fracture repair, and it may have negative effects on new bone formation in distraction osteogenesis. The treatment of type II diabetes with Rosiglitazone may have further negative effect on bone formation [16], while treatment with Metformin may have positive effects [17].

Disorders affecting general nutrition and intestinal absorption, such as anorexia [18] and celiac disease [19] can profoundly affect bone health, and negatively influence osteogenesis.

Several medications can negatively affect bone repair. This does not automatically imply that bone formation in distraction osteogenesis is reduced, because the details of fracture repair and distraction osteogenesis are not identical. Corticosteroids have not shown consistent negative effects on fracture repair, although most studies point in that direction. In rats, endochondral bone formation was negatively effected, but intramembranous bone formation was not. It may be, therefore, that the negative effect on bone formed during distraction osteogenesis is minor [20]. Chemotherapeutic agents in general may have some negative effects on bone healing, but in experimental and clinical studies on distraction osteogenesis to reconstruct surgical defects, mostly positive results have been reported [20, 21]. The antibiotics mostly used in conjunction with distraction osteogenesis appear to be harmless with regards to bone formation [20]. Prophylactic use of low molecular weight heparins appear to have no negative effect on fracture healing, but antithrombotic therapy using heparin decreases bone formation and increases resorption [20]. The effect of NSAIDs is still somewhat controversial. However, drugs with a short serum half-life, e.g. ibuprofen, appear to have little negative effect on fracture healing and osteogenesis when given short term and in moderate dose. Indomethacin appears to be an exception [20, 22].

For a variety of reasons, patients exhibiting chronic substance abuse are considered poor candidates for complicated orthopaedic procedures. Chronic alcoholism is known to be associated with osteoporosis and impaired fracture repair. Distraction osteogenesis in this setting has not been studied clinically, but animal experiments show a profound negative effect of chronical alcohol intake [23]. Smoking has a negative influence on bone healing, including bone formation in distraction osteogenesis [24].

## Treatment of deficient bone formation

Prior to lengthening procedures, it is advisable to investigate serum 25-OH vitamin D which should not be below 25 nmol/l, and preferably be 75–150 nmol/l. An adequate daily supply of mineral, primarily calcium, should be ensured. In general, it is better to anticipate problems with bone formation than to have to manage a deficient regenerate or an atrophic non-union later on.

Patients with congenital shortenings are at higher risk of deficient bone formation, reflected in a higher risk of fracture after fixator removal. The fracture risk was higher in these patients when lengthening was >15% of initial segment length (except for patients with achondroplasia), and when the latency period was less than 7 days [25]. Bone fragility is present in more than 100 different genetic disorders including skeletal dysplasias [26]. Among these, achondroplasia patients are by far the most common candidates for limb lengthening procedures, and they are known to tolerate these procedures relatively well. Another, much more difficult patient group is neurofibromatosis (and congenital pseudarthrosis not related to this disorder). Other osteochondrodysplasias are relatively rare candidates for limb lengthening procedures, and clinical experience accordingly limited.

Bone formation may be deficient in terms of volume and quality. If volume is insufficient, a change of the lengthening protol, reducing the number of increments per day (and thus the amount of daily lengthening), and in more severe cases intermittent compression–distraction (accordion maneuvre), can be attempted. If volume is still deficient by the end of the lengthening procedure, the first line of treatment would be autologous bone grafting. If quality is deficient reflecting reduced mineral content of the newly formed bone, vitamin D status should be checked. This condition of protracted consolidation will postpone frame removal beyond the average. The patient should be physically active and bear full weight on the limb [14]. Submuscular plating or intramedullary nailing may be considered to reduce time in fixator, but these measures involve a significant risk of infection when performed after a period of external fixation. Motorized nailing is now being used in older children and adults and may reduce the problem of delayed mineralization to some extent. Lengthening over one or two flexible nails may be considered, and has been shown to reduce the time on external fixation [27].

Bone transport procedures add the challenge of healing of the docking site. In some instances, this may be circumvented by acute shortening after bone resection, then lengthening. Docking would usually involve planned autologous bone grafting, or other means of stimulation of local repair.

Mechanotransduction, i.e. the promotion of bone formation by mechanical means, can possibly be applied effectively by other means than weight bearing and frame manipulations. Low intensity pulsed ultrasound and pulsed electromagnetic fields can deliver strains in the micro-range to the regenerate during and after distraction. The efficacy of pulsed ultrasound in shortening the treatment time in tibial lengthening is documented in randomized clinical trials [28]. Femoral lengthening would not appear to be a target, since the soft tissue envelope does not transmit the ultrasound waves effectively. There is some documentation that pulsed electromagnetic fields may have clinical value, including an internally controlled study (bilateral lengthening, one side treated) in 30 patients, most of them achondroplasia patients. The treated side showed earlier callus formation and maturation, and the frame could be removed about 1 month earlier on the treated side [29].

## Stem cells and platelets

There is no doubt that MSCs are important for successful fracture healing and distraction osteogenesis. Platelets contain multiple growth factors important in bone regeneration. Both are relatively readily available for autologous use, and may be concentrated by centrifugation during surgery. The cell and platelet contents in the crude aspirate and after centrifugation may vary. This may be of minor importance for the individual patients, but cell (platelet) counts and cell characterization are of importance in clinical research, since success or failure may depend on the number and types of cells injected. Failure to provide such information may jeopardize the conclusions made, and makes comparison of studies difficult or impossible. The procedures involved in collecting marrow and blood during a surgical procedure and the following centrifugation and aspiration of cell concentrates are relatively safe with little risk involved of contamination or other potential danger to the patient. More sophisticated procedures, involving isolation and cultivation of cells outside the operating room obviously present considerably more risks.

While there is abundant literature on experimental animal research to document the potential of marrow derived stem cells and platelets to enhance bone formation in distraction osteogenesis, the clinical literature is sparse. A recent randomized clinical trial with ten patients in each group reports on bilateral tibial lengthening for familial short stature. The treatment group had injection locally in the osteotomy of a mixture of centrifugated marrow aspirate and platelet rich plasma obtained and injected during the index surgical procedure. There was no difference in the time in external fixator between the groups, but the cortical healing index was about 15% less in the treatment group as was the time to full weight bearing [30]. These cells were injected into the osteotomy at the time of surgery. Cells may be injected later in the process of lengthening, e.g. to boost an insufficient regenerate during or after distraction.

## Growth factors

Two growth factors are commercially available, BMP-2 (InductOs®, Infuse®) and BMP-7 (also known as osteogenic protein-1 (Osigraft®). These GF’s are approved for use in lumbar spondylodeses and tibial fractures, and tibial non-unions, respectively. They are not approved for use in children due to concerns about cancer risk. Large cohort studies conclude that the use of BMP-2 in lumbar spondylodeses in an elderly population is not associated with increased cancer risk, but the issue remains controversial [31]. Off-label use in children is possible. BMP-2 has been used in children undergoing scolioses surgery and lower extremity surgery. Such use should be cleared with national health authorities. The use of growth factors in connection with distraction osteogenesis would mostly be indicated to augment healing potential of bone grafting in deficient callus and docking site non-union.

## Systemic medications

Bisphosphonates and PTH are used to treat osteoporosis. Bisphosphonates (BP) are incorporated into bone. Osteoclasts ingesting this bone undergo apoptosis. Thus, BP’s inhibit bone resorption. BP’s are used off-label to treat children with osteogenesis imperfecta. In distraction osteogenesis, the volume of the regenerate bone increases as do the mechanical strength, and native bone is protected from disuse osteopenia. Successful use in children has been reported, BP being given during the consolidation phase to rescue regenerate insufficiency [32]. BP’s would be expected to be effective when catabolic regenerate failure is present, and not when anabolic failure is predominant. Short term treatment does not have long-term effects on remodelling, and therefore the risk of complications related to long-term and high-dose use (atypical long-bone fracture, osteonecrosis of the jaw) can be considered minimal.

PTH is used clinically to treat osteoporosis in a preparation known as teriparatid [PTH (1–34)], the N-terminal of the native human hormone (Forsteo®). It has not been used clinically in distraction osteogenesis, and only sporadic use in orthopaedic conditions have been reported. In animal studies on distraction osteogenesis, PTH has shown significant anabolic effects. Since the effect is almost as large when given during the consolidation phase as compared to the distraction and consolidation phases, it holds promise as a rescue medication for the anabolic insufficient regenerate [33]. The use in orthopaedic conditions is off-label. Due to possible cancer risk with long-term use (animal studies), the duration of treatment should not exceeded 24 months over a patients life time. It should not be given during pregnancy, and it should not be given to children or adolescents with open epiphyses.
